# An Aquaporin 3-Notch1 Axis in Keratinocyte Differentiation and Inflammation

**DOI:** 10.1371/journal.pone.0080179

**Published:** 2013-11-08

**Authors:** Liqiong Guo, Hongxiang Chen, Yongsheng Li, Qixing Zhou, Yang Sui

**Affiliations:** 1 Key Laboratory of Pollution Processes and Environmental Criteria, College of Environmental Sciences and Engineering, Nankai University, Tianjin, China; 2 Department of Dermatology, Union Hospital, Tongji Medical College, Huazhong University of Science and Technology, Wuhan, Hubei, China; 3 Cutaneous Biology Research Center, Department of Dermatology, Massachusetts General Hospital, Harvard Medical School, Boston, Massachusetts, United States of America; 4 Center for Experimental Therapeutics and Reperfusion Injury, Harvard Institutes of Medicine, Department of Anesthesiology, Perioperative and Pain Medicine, Brigham and Women’s Hospital and Harvard Medical School, Boston, Massachusetts, United States of America; 5 Department of Bioinformatics, International School of Software, Wuhan University, Wuhan, Hubei, China; University of Tennessee, United States of America

## Abstract

Aquaporin 3 (AQP3) is an aquaglyceroporin which transports water, glycerol and small solutes across the plasma membrane. Its functions are not limited to fluid transport but also involve the regulation of cell proliferation, migration, skin hydration, wound healing and tumorigenesis. While AQP3 has been reported to play an important role in keratinocyte proliferation, its role in differentiation remains controversial. Our study demonstrated that the expression of AQP3 was regulated during differentiation and that it participated in keratinocyte differentiation control. We further revealed that AQP3 was a transcriptional target of Notch signaling, a critical pathway regulating keratinocyte differentiation and tumor suppression, and it regulated differentiation through a reciprocal negative feedback loop with Notch1. When the expression level of AQP3 was elevated, impaired barrier integrity and increased pro-inflammatory cytokine production ensued, mimicking the pathological conditions in Notch deficient mice and in atopic dermatitis. Dysregulation of AQP3 and Notch receptors has been reported in several skin diseases, including skin cancer. Our discovery of the novel AQP3-Notch1 axis may provide insight into epidermal homeostasis control and possible translational applications, including its potential use as a biomarker for molecular diagnosis in environmental studies.

## Introduction

The aquaporins are a family of transmembrane channels transporting water and in some cases, small solutes across the plasma membrane driven by osmotic gradients [1]. Currently, 13 mammalian aquaporins have been identified (AQP0-AQP12). They are expressed in tissues involved in active fluid transport such as kidney tubules, secretory epithelia and microvascular endothelia as well as tissues without significant fluid transport, including the epidermis, skeletal muscle, adipose tissue, erythrocytes and leukocytes [1]. Studies from aquaporin-null mice revealed important functions of aquaporins in urine concentration [2,3], brain water balance [4,5] and neuroexcitation [6–8], corneal transparency and retina swelling [9,10], skin hydration and elasticity [11,12], cell migration [13–16], cell proliferation [14,17], wound healing [15], angiogenesis [13] and fat metabolism [18].

Aquaporin-3 (AQP3) is the predominant and most widely studied aquaporin in mammalian skin. It is an aquaglyceroporin and is capable of transporting water, glycerol, urea and hydrogen peroxide [1]. The functions of AQP3 in skin are mostly concluded from studies in AQP3-deficient mice. AQP3-mediated glycerol transport plays an important role in stratum corneum hydration, skin elasticity, barrier recovery, wound healing and cell proliferation whereas AQP3-mediated water transport is critical for cell migration [11,15]. Recently, AQP3 was reported to mediate hydrogen peroxide uptake which was responsible for regulation of T-cell migration and cutaneous immune response [19,20]. The above functions of AQP3 may explain, in part, the resistance of AQP3-deficient mice to epidermal tumor formation in chemical carcinogen-induced tumorigenesis [21]. The homeostasis of mammalian skin, especially the epidermis, is maintained by tightly regulated proliferation/differentiation. Despite its known function in keratinocyte proliferation, the role of AQP3 in keratinocyte differentiation remains controversial. 

Notch signaling is an evolutionarily conserved cell-cell communication pathway and plays a critical role in various physiological and pathological processes [22]. There are four vertebrate Notch receptors, Notch1-4. The Notch proteins are type-1 trans-membrane receptors, upon interaction with membrane-bound ligands, such as delta-like, or Jagged on neighboring cells, the receptors will undergo two proteolytic cleavages to become activated. The first cleavage is mediated by metalloproteases, ADAM10/TACE/Kuz/SUP-17, in the extracellular portion of the Notch C-terminus. This cleavage produces the membrane–anchored Notch extracellular truncation (NEXT) which is subsequently cleaved by intramembrane aspartyl protease complex γ-secretase, leading to the release of Notch intracellular domain (NICD) from the plasma membrane. The NICD will then translocate to the nucleus, displace corepressor complexes associated with DNA-binding protein CSL and form a transcriptional complex with CSL and coactivators to activate the transcription of Notch target genes [23]. The best characterized Notch signaling targets are members of the HES and HERP families of bHLH transcriptional repressors [24]. In mammalian skin, Notch1-mediated signaling exerts a critical pro-differentiation and tumor suppressing function [25–27]. 

Using gain and loss of function approaches, we demonstrated that AQP3 regulated differentiation through a reciprocal negative feedback loop with Notch signaling. Its increased expression, as observed in atopic dermatitis, led to decreased expression of an important epidermal barrier component, filaggrin, and increased expression of pro-inflammatory cytokines, including TNFα and CCL5. The interconnection between AQP3 and Notch1 may have general relevance in the physiological and pathological processes of the skin.

## Results

### 1): The expression of AQP3 decreases during late differentiation in human keratinocytes concurrent with Notch signaling activation

**Figure 1:**
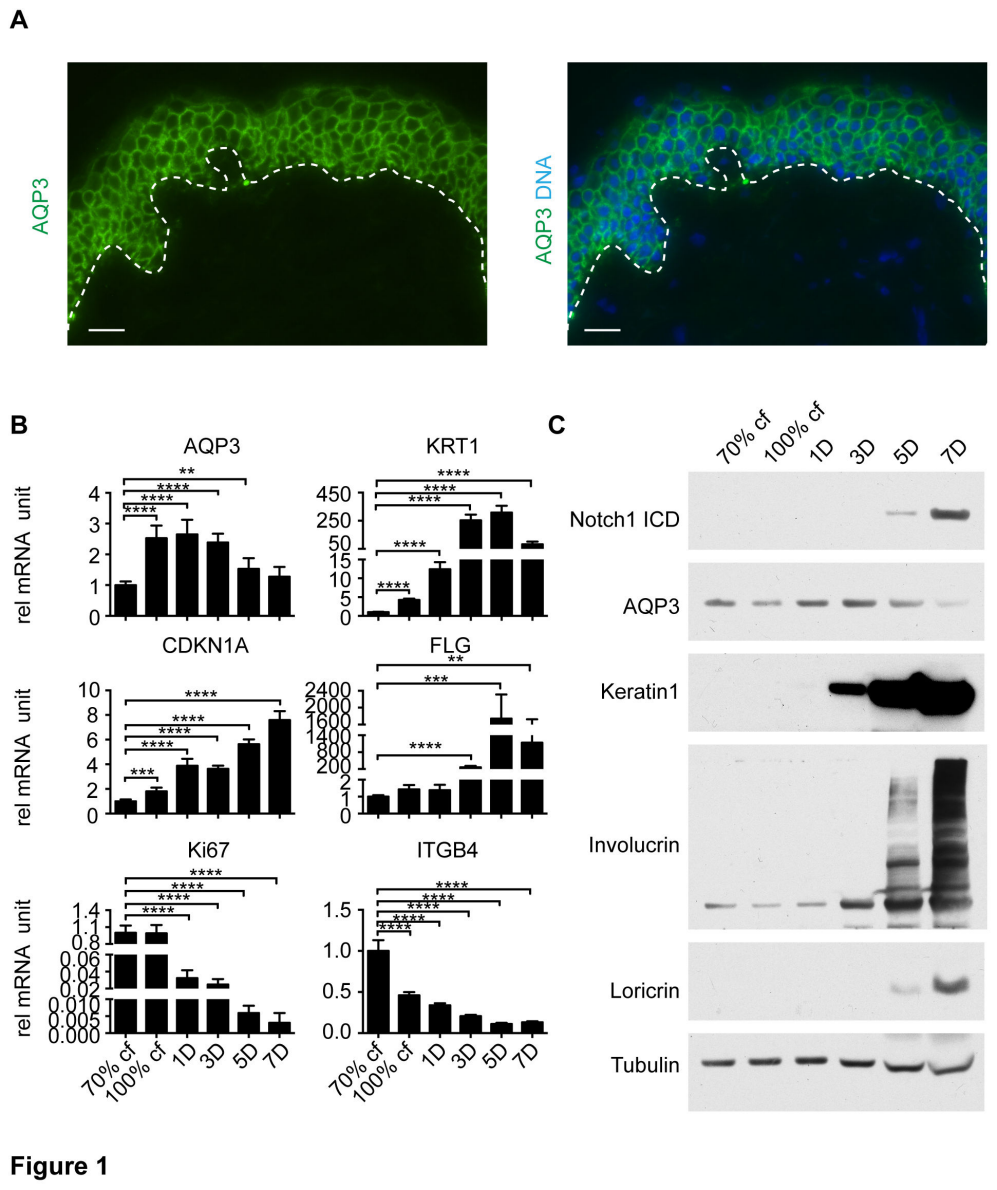
AQP3 expression is down-regulated during late differentiation concurrent with Notch signaling activation. (A) Immunofluorescence analysis of AQP3 expression in normal human skin. Frozen sections (8 μm) of normal human skin were stained with an antibody against RORα (green) and Hoechst (blue) for DNA. Images are representatives of several independent fields. Bar=100 μm. (B) qRT-PCR analysis of expression of the indicated genes in HKCs under proliferating condition (70% confluence, cf) and at various time (days, D) of differentiation induced by high cell density. Values are normalized to reference ribosomal gene RPLP0, and presented as fold-changes relative to cells under proliferating condition ± S.E.M. n=3. ** p< 0.01, *** p < 0.001, **** p<0.0001. (C) Immunoblot analysis of indicated proteins in the same time course experiment as in (B).

To determine the potential involvement of AQP3 in keratinocyte differentiation, we first examined whether the expression of AQP3 was regulated during differentiation. Consistent with previous studies, immunofluorescence staining of intact human skin showed that AQP3 was mostly expressed on the plasma membrane of keratinocytes in both basal and spinous layers of the epidermis with decreased expression towards the stratum corneum [28,29] (Figure 1A). In parallel, the expression of AQP3 was evaluated in cultured primary human keratinocytes (HKCs) under proliferating condition or upon induction of differentiation by high cell density [30,31]. The expression of markers characteristic of basal (integrin β4), spinous (keratin 1 and involucrin) and granular (loricrin and filaggrin) layers, proliferation marker (Ki67) and cell cycle inhibitor p21^WAF1/Cip1^ (*CDKN1A*) was assessed to verify proliferation and differentiation progression. The results showed that high cell density led to a decrease in the expression of Ki67 and integrin β4 and an increase in *CDKN1A* and all of the differentiation markers (keratin 1, involucrin, loricrin and filaggrin), reflecting differentiation induction and accompanying cell cycle exit (Figure 1B, C). Quantitative real-time PCR and immunoblot analysis showed that AQP3 was expressed at all cell densities: pre-confluent (70% confluent), confluent (100% confluent) and post-confluent (1-7 days after reaching confluence), with slight increase in early post-confluent time points (1D and 3D) and sharp decrease in late time points (5D and 7D) (Figure 1B, C). AQP3 expression increased during early differentiation when keratin 1 started to appear and decreased during late differentiation when loricrin began to be expressed (Figure 1B, C). Interestingly, the decrease in AQP3 expression at post-confluent day 5 and 7 coincided with an increased expression of activated Notch1(N1ICD) (Figure 1C). Given the known role of Notch signaling in keratinocyte differentiation [25,26,32], the inverse expression pattern of N1ICD and AQP3 suggested that AQP3 might be negatively regulated during differentiation by Notch signaling. 

### 2): AQP3 is under the negative regulation of Notch signaling

**Figure 2:**
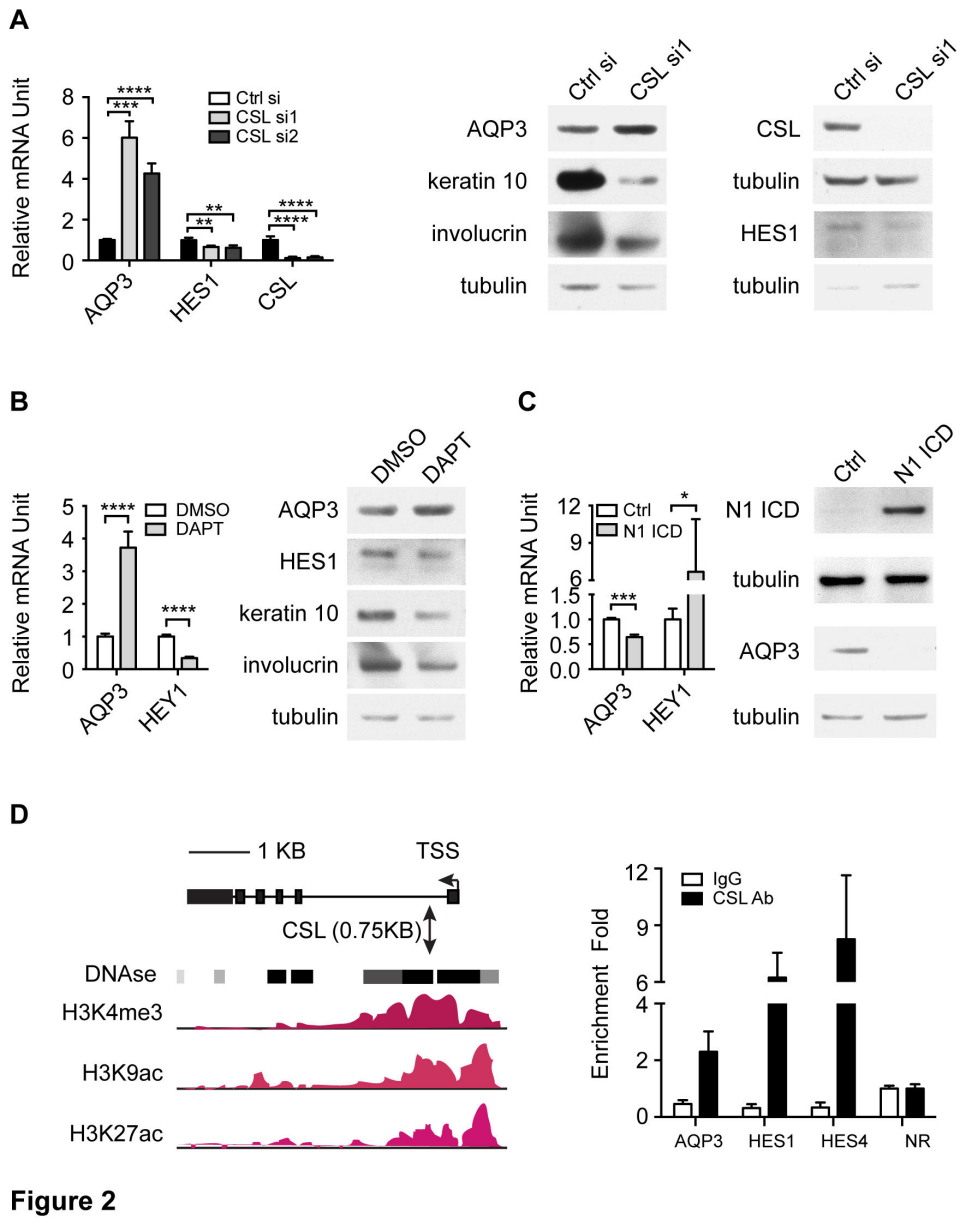
AQP3 is a transcriptional target of Notch signaling. (A) HKCs reverse transfected with siRNAs against CSL or scrambled control were analyzed 1 week later (in differentiating condition) by qRT-PCR and immunoblot of the indicated genes and proteins. mRNA levels were normalized to reference ribosomal gene RPLP0, and presented as mean fold-change over control ± S.E.M. n=3. ** p< 0.01, *** p < 0.001, **** p<0.0001, Hes1, Keratin 10 and Involucrin were used as indicators of endogenous Notch activity, γ-tubulin served as loading control. (B) Differentiating HKCs were treated with DAPT (10μM) or DMSO control for 72 hours followed by qRT-PCR and immunoblot analysis of the indicated genes and proteins. The analysis was done as in (A). **** p<0.0001, n=3. (C) Proliferating HKCs were infected with retroviruses expressing activated Notch1 or GFP followed, 72 hours later, by qRT-PCR and immunoblot analysis of AQP3. Hey1 was used as an indicator of endogenous Notch activity. The analysis was done as in (A). * p<0.05, *** p<0.001, n=3. (D) Chromatin immuno-precipitation (ChIP) analysis of CSL binding to the regulatory region of the *AQP3* gene. Left panel: schematic representation of the *AQP3* gene locus based on ChIP-seq information on different chromatin states as provided by the ENCODE project. The location of CSL binding site in the *AQP3* gene was analyzed using Mat-inspector software. Right panel: HKCs were processed for ChIP analysis using CSL antibody or non immune IgG. Results are expressed as mean fold of enrichment ± S.E.M. n=2, for each indicated binding site, after immunoprecipitation with CSL antibody or non immune IgG, relative to enrichment for the negative control region.

To assess whether AQP3 was under Notch signaling control, we evaluated its expression in HKCs upon either Notch signaling inactivation by CSL knockdown or γ-secretase inhibition, or activation of the pathway by overexpression of activated Notch1. In differentiating HKCs where Notch signaling is activated, Notch signaling inactivation by CSL knockdown led to a reduced expression of Notch canonical target HES1 and differentiation markers. However, the expression of AQP3 increased at both mRNA and protein levels (Figure 2A). Similar induction of AQP3 was observed in differentiating HKCs treated with DAPT, a γ-secretase inhibitor which suppresses endogenous Notch activation (Figure 2B) [33]. In contrast, constitutive expression of activated Notch1 in proliferating HKCs by retroviral infection resulted in a reduction of AQP3 expression, whereas HEY1, another canonical target of Notch signaling was greatly induced (Figure 2C). To substantiate our findings, bioinformatic analysis was performed to search for putative CSL binding sites located within potential transcription regulatory regions of the AQP3 gene utilizing chromatin configuration information provided by the ENCODE project (http://genome.ucsc.edu/ENCODE/). The motif search identified a consensus CSL binding site located about 750bp downstream of transcription start site (TSS) of the AQP3 gene in a region enriched for active promoter marks, including H3K4me3, H3K9ac and H3K27ac and with DNase I hypersensitivity. Chromatin immunoprecipitation (ChIP) assay was performed with extract from HKCs under high cell density differentiating condition using CSL antibody. In differentiating HKCs, endogenous CSL bound to its consensus binding site located in the AQP3 gene promoter in parallel to its binding to the promoters of HES1 and HES4 genes, two canonical targets of Notch signaling, whereas no binding was detected in α satellite repeat element located in the pericentromeric regions of chromosomes, indicating that AQP3, like HES1 and HES4 was under direct transcriptional control of Notch signaling (Figure 2D). 

### 3): AQP3 inhibits keratinocyte differentiation through down-modulating Notch1 expression

**Figure 3:**
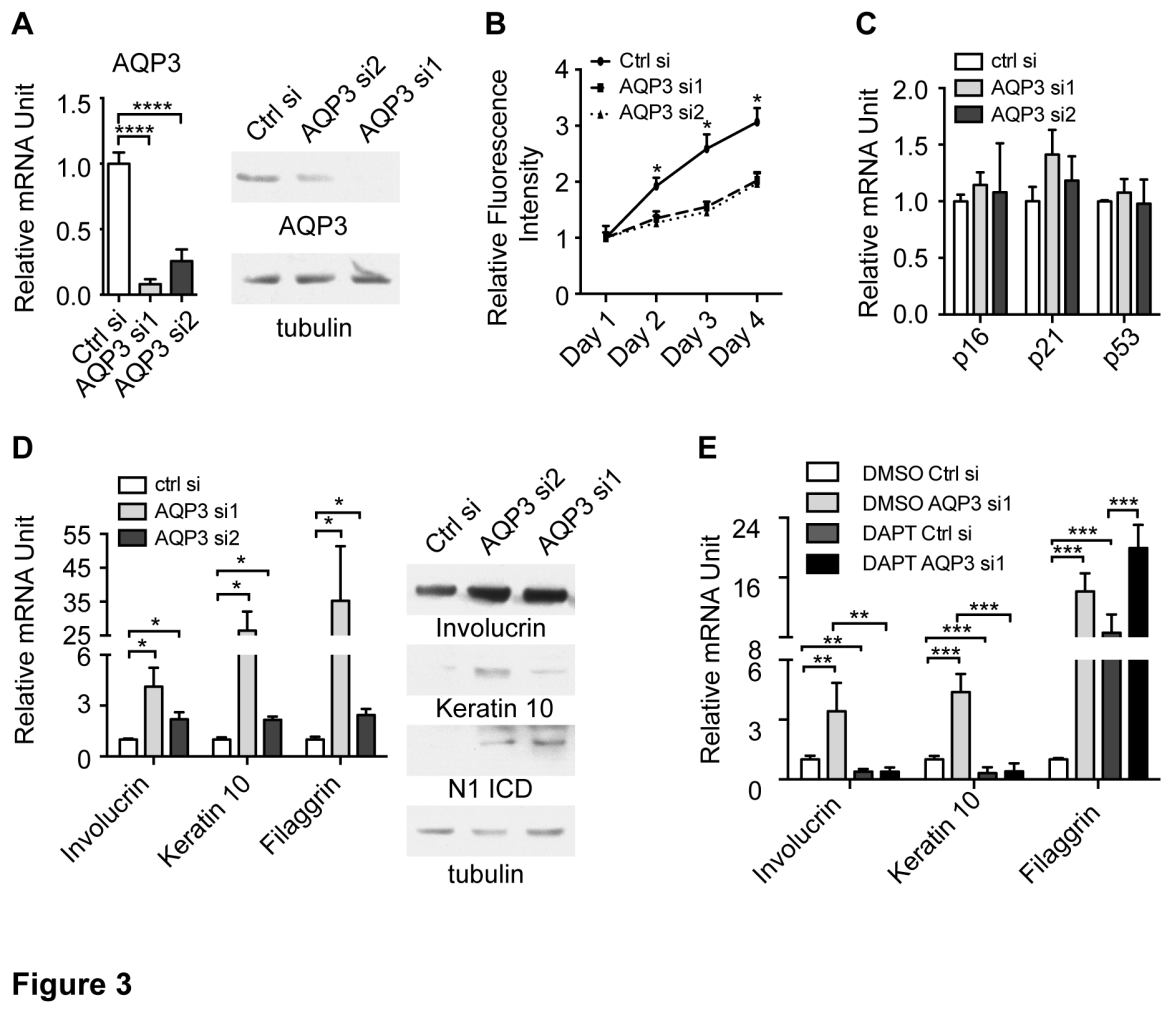
Attenuated AQP3 expression promotes differentiation through activating Notch1. (A) HKCs reversely transfected with siRNAs against AQP3 or scrambled control were analyzed 72 week later (in proliferating condition) by qRT-PCR and immunoblot of AQP3. The analysis was done as in Figure 2A. **** p<0.0001 n=3. (B) Alamar blue cell proliferation assay of HKCs with AQP3 silencing. HKCs reverse transfected with siRNAs against AQP3 or scrambled control as in (A). 48 hours post-transfection, cells were trypsinized and plated in 96-well plate at a density of 1000 cells/well in triplicate. Fluorescence intensity was measure for four consecutive days. Data are presented as mean fold change of fluorescence intensity ± S.E.M. over day 1. * p<0.0001, n=3. (C) Same samples as in (A) were analyzed by qRT-PCR of the indicated genes. (D) Same samples as in (A) were analyzed by qRT-PCR and immunoblot of the indicated genes and proteins. * p<0.0001, n=3. (E) HKCs were reverse transfected with siRNAs against AQP3 or scrambled control, followed by 48 hours later, incubation with DMSO or DAPT (10μM) for additional 3 days. The expression of indicated genes were analyzed by qRT-PCR. Results are presented as mean fold-change over control ± S.E.M. n=3. One-way ANOVA was used for statistical analysis. ** p= 0.0023, *** p=0.0001.

The negative regulation of AQP3 by Notch signaling suggested that down-modulation of AQP3 might be required for normal differentiation. To evaluate the functional significance of AQP3 expression, we used gain and loss of function approaches to manipulate AQP3 level in HKCs. In loss of function approach, two individual validated siRNA for AQP3 were used to silence AQP3 expression in HKCs under proliferating condition (Figure 3A). In agreement with previous reports[21], attenuated AQP3 expression caused growth inhibition (Figure 3B). However, the expression of cell cycle regulators, including p21^WAF1/Cip1^, and p16^Ink4A^ or apoptosis regulator p53 was not affected by AQP3 silencing, suggesting the growth inhibition observed was likely due to other mechanisms (Figure 3C). In contrast to previous reports [29], AQP3 silencing led to an increased expression of early markers such as keratin 10 and involucrin at both mRNA and protein levels as well as an increase in filaggrin mRNA, a gene normally being transcribed during late differentiation (Figure 3D). Of note, the level of activated Notch1 was significantly increased upon AQP3 silencing, pointing to the possibility that AQP3 silencing-induced differentiation could be the consequence of activated Notch signaling (Figure 3D). To test this hypothesis, HKCs transfected with AQP3 siRNAs were cultured in the presence or absence of DAPT and the expression of differentiation markers was evaluated. In the presence of DAPT, the induction of keratin 1 and involucrin was mostly abrogated, confirming that their induction by AQP3 was through Notch activation (Figure 3E). Filaggrin has been reported to be under the negative regulation of Notch signaling [26], AQP3 silencing caused a significant induction of filaggrin expression in HKC which was slightly augmented by DAPT treatment, suggesting the induction of filaggrin by AQP3 was independent of Notch signaling (Figure 3E). 

**Figure 4:**
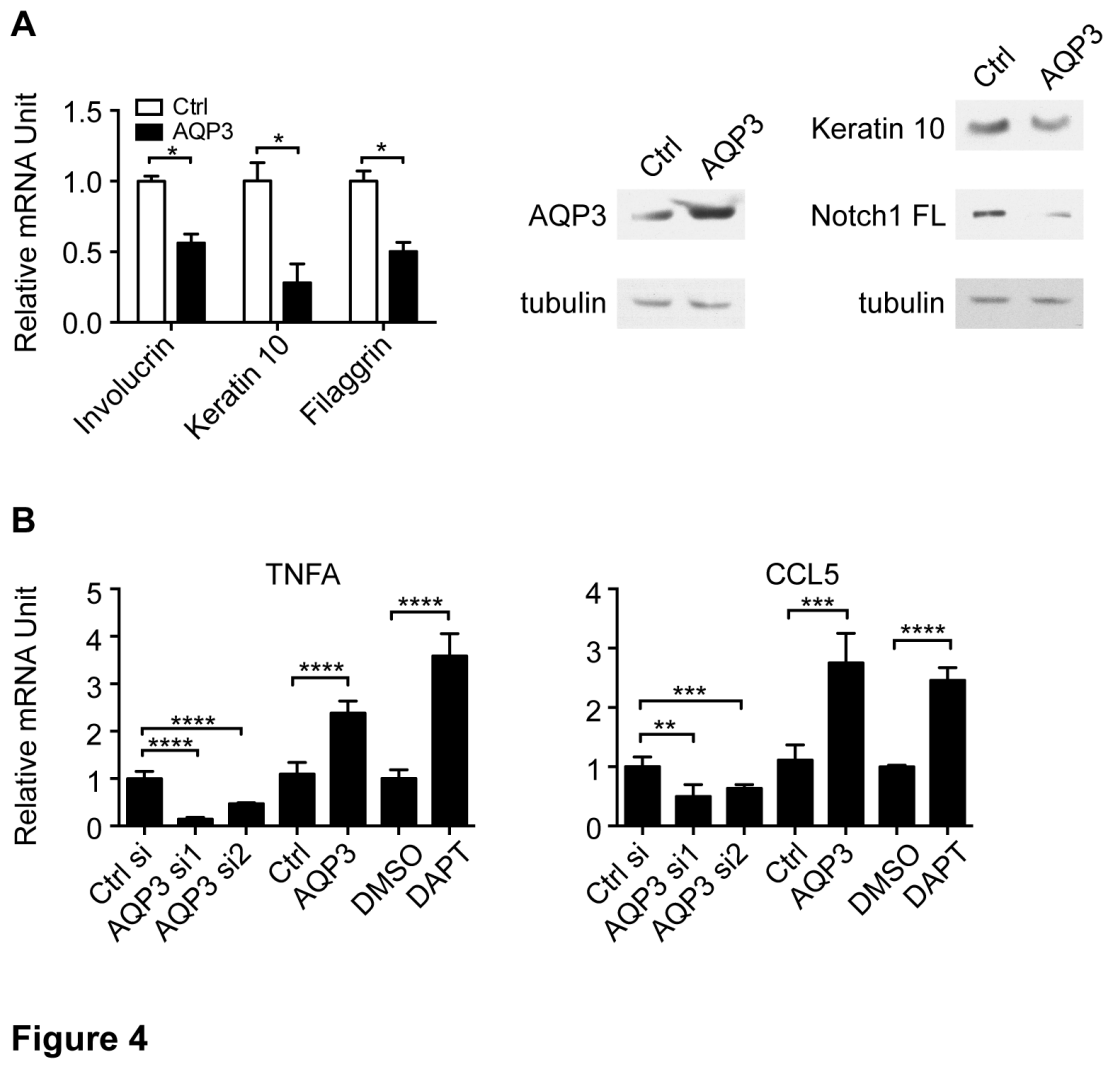
Increased AQP3 expression suppresses the expression of differentiation markers and induces the expression of pro-inflammatory cytokines. (A) HKCs were infected with retroviruses expressing AQP3 or GFP followed, 72 hours later (differentiating condition), by qRT-PCR and immunoblot analysis of the indicated genes and proteins. The analysis was done as in Figure 1. * p< 0.0001, n=3. (B) HKCs were either reverse transfected with siRNAs against AQP3 or scrambled control and analyzed 96h later (differentiating condition) or infected with retroviruses expressing AQP3 or GFP and analyzed 72 hours later (differentiating condition) by qRT-PCR analysis of the indicated genes. In parallel, differentiating HKCs were treated with DAPT (10μM) or DMSO control for 72 hours followed by qRT-PCR analysis of the indicated genes. The analysis was done as in Figure 1. ** p< 0.01, *** p < 0.001, **** p<0.0001, n=3.

In the converse experiments, overexpressing AQP3 in differentiating keratinocytes led to a reduced expression of involucrin, keratin 10, filaggrin as well as Notch1 (Figure 4A). In summary, the gain and loss of function studies demonstrated that AQP3 negatively regulated differentiation possibly through its interconnection with Notch signaling.

### 4): Increased AQP3 expression results in impaired epidermal barrier and pro-inflammatory cytokine production

Increased expression of AQP3 has been reported in atopic dermatitis, one of the most common inflammatory skin diseases [34,35]. An independent line of studies has found that Notch deficient epidermis exhibited atopic dermatitis-like phenotype [36,37]. To determine whether AQP3 played a role in the inflammatory response, we surveyed the expression of a number of pro-inflammatory cytokines upon AQP3 silencing or overexpression in comparison to Notch signaling inhibition by DAPT treatment in HKCs. The results showed that among the factors tested, TNFα and CCL5 were consistently modulated in these various conditions: AQP3 silencing down-regulated whereas AQP3 overexpression and Notch signaling inhibition up-regulated their expression (Figure 4B). Together with its capability of suppressing filaggrin expression, one of the important proteins in epidermal barrier, and differentiation markers, AQP3 overexpression led to barrier defect and pro-inflammatory cytokines production. 

## Discussion

Epidermal homeostasis is maintained by a fine balance between proliferation and differentiation. AQP3-mediated water and glycerol transport play an important role in a number of cellular and physiological processes in the epidermis, such as cell migration, proliferation, hydration, wound healing and tumorigenesis [38]. Our investigation of AQP3’s involvement in keratinocyte differentiation and its interconnection with Notch signaling was ignited by the conflicting results in the literature as well as the inverse expression patterns of Notch receptors and AQP3 in skin diseases [29,36,39,40]. We showed that AQP3 was expressed not only in proliferating keratinocytes but also cells undergoing early differentiation. We further demonstrated that the down-modulation of AQP3 during late differentiation was concurrent with Notch activation and that AQP3 was under the negative regulation of Notch. In proliferating and early differentiating keratinocytes, AQP3 suppressed differentiation and promoted proliferation through down-regulation of Notch1. Our study supports the notion that AQP3 regulates keratinocyte differentiation and identified Notch signaling as the mediator of this function.

Previously, Hara-Chikuma et. al. reported that AQP3 was not involved in keratinocyte differentiation using AQP3 null mice and neonatal human keratinocytes [29]. In contrast, it was suggested by Bollag et. al. that AQP3 participated in keratinocyte early differentiation regulation by regulating lipid synthesis [39]. Kim and Lee also demonstrated that attenuated expression of AQP3 led to a decreased expression of keratin 10 in response to calcium induced differentiation [41]. The disagreement between our findings and other reports could be due to several possibilities. First, in mouse skin, AQP3 expression is restricted to the proliferating basal layer whereas in human skin, it is expressed in both basal and differentiating spinous layers [12,28]. The difference in expression patterns of AQP3 suggests human AQP3 may have acquired new functions in differentiating cells. Second, technical differences may create the discrepancy. In our study, we assessed the effect of AQP3 silencing in proliferating keratinocytes when it was highly expressed and found that AQP3 attenuation led to increased expression of differentiation markers when their expression were supposed to be low or undetectable. We also extended the siRNA study to one week time point when keratinocytes underwent early differentiation, the effect of AQP3 on differentiation markers persisted (data not shown). In other AQP3 silencing studies, high concentration calcium was used to induce differentiation during a lengthy period of time. Various differentiation inducing agents, such as calcium, PMA and 1,25-dihydroxyvitamin D3 induce differentiation through different or partially overlapping mechanisms [42]. It may be possible that the effect of AQP3 on differentiation was counteracted by calcium. In addition, the effect of AQP3 siRNA may be lost after prolonged experimental procedure and thus lead to a misleading conclusion. Moreover, primary keratinocytes have very low transformation efficiency. In our study, we used high titer retrovirus to achieve homogenous expression of the transgene and to eliminate the effect from uneven transfection. Finally, experimental time points in our investigation were selected to best reflect and evaluate the physiological impact. For example, due to its association with corepressor complexes, there are two possible consequences of CSL silencing. First, if it occurs in the absence of Notch activation, Notch targets which are normally repressed by CLS could be activated. Second, if CSL is silenced in parallel with Notch receptor activation, Notch signaling will be significantly impaired. In our study, the effect of CSL silencing on AQP3 was examined at a time point (1 week post transfection) when Notch was activated (Figure 1C) and it represented a Notch signaling deficiency condition.

Notch signaling plays a critical role in epidermal homeostasis by promoting terminal differentiation to maintain epidermal barrier integrity, mediating communication between epithelial and immune cells to prevent inflammation and tumorigenesis [27,36,43]. The identification of AQP3-Notch1 interconnection suggests that Notch signaling may play a role in epidermal water homeostasis and lipid biosynthesis. It has been reported that deficiency of Fbxw7, a negative regulator of Notch signaling, leads to massive lipid deposition in the liver, a phenotype which can be corrected by Notch inhibition [44]. It would be informative to evaluate whether there is any change in glycerol level, transepidermal water loss and lipid content and composition in the epidermis of Notch null mice. The reciprocal effect of AQP3 on Notch1 was limited to protein level, suggesting the effect was post-transcriptional. Interestingly, a recent study by Roti et. al. demonstrated that calcium channels are required for the maturation of oncogenic Notch1 in T cell acute lymphoblastic leukemia [45]. It would be of great interest to see how AQP3 mediated water/glycerol transport regulates Notch1 protein expression. 

Dysregulated expression of AQP3 and Notch receptors have been reported in a number of skin diseases, including atopic dermatitis [34–36], psoriasis [46,47] and skin cancer [21,27,48]. Most studies demonstrated an inverse expression pattern of AQP3 and Notch1, supporting the reciprocal negative feedback loop identified in our study. We showed that elevated expression of AQP3 led to a reduced expression of differentiation markers, filaggrin in particular and an increased expression of CCL5 and TNFα, two of the most prominent cytokines in atopic dermatitis. Moreover, the changes induced by AQP3 mirrors the effect of Notch inhibition, suggesting AQP3 might not play a passive role in the development of atopic dermatitis.

The altered distribution and expression of aquaporins (AQP3) in pathological conditions have led to the growing consensus that modulation of AQPs functions may be therapeutically useful. A number of factors have been implicated in AQP3 regulation, including PPARγ [49], TNFα [50], retinoic acid [51,52], EGFR [53], calcium [42] and low pH [12]. The identified Notch1/AQP3 interconnection provides a regulatory mechanism for both factors. In the light that their inverse expression pattern is present in several skin diseases, it is worth evaluating whether the Notch1/AQP3 functional axis is present in those pathological conditions. Further characterization of this interconnection may be of great potential therapeutic use. 

## Materials and Methods

### Ethics Statement

The use of discarded human skin tissues for keratinocyte isolation was specifically approved by the Medical Ethics Committee of Tongji Medical College, Huazhong University of Science and Technology (Ethics approval No. 2011S007 and 2012S052). The committee waived the need for written informed consent from patients.

### Keratinocyte preparation and cell culture

Primary human keratinocytes (HKC) were isolated from discarded skin samples from abdominoplasty procedures with patients’ and institutional approvals. Briefly, connective tissue was removed and the skin was incubated overnight in 10mg/ml dispase at 4°C. The epidermis and dermis were separated and keratinocytes were collected from 0.025% trypsin digested epidermis. HKCs were cultured in serum-free keratinocyte-SFM medium (Gibco) supplemented with 30 μg/ml bovine pituitary extract (BPE), 0.1 ng/ml rEGF and antibiotics (Gibco) on collagen coated dishes.

### Plasmids and retrovirus

The expression vector for activated Notch1 and corresponding control vector were kindly provided by Dr. U Just (Christian-Albrechts-University of Kiel, Germany)[54]. pMSCV-AQP3 was constructed by cloning the Flag-tagged full-length cDNA of AQP3 into the XhoI/EcoRI sites of the pMSCV-puro vector using the following primers: forward 5’-GATTCCCTCGAGGCCACCATG GACTACAAGGACGACGATGACAAGGGTCGACAGAAGGAGCTGGTG-3’ and reverse 5’-GCT GCTGAATTCCTAGATCTGCTCCTTGTGCTTTCAGATCTGCTCCTTGTGCTT-3’. Conditions for retrovirus production and infection were as previously reported [55].

### Quantitative real time PCR and Immunodetection

For mRNA analysis, 400 ng of total RNA, isolated with RNeasy Mini QIAcube kit (Qiagen), was reversely transcribed using the iScript cDNA synthesis kit (Bio-Rad). Real time PCR with SYBR Green detection was performed on the Light Cycler 480 instrument, according to manufacturer’s instructions (Roche). Each sample was prepared in triplicate, and results were normalized with the expression of the housekeeping 36β4 gene. The list of gene-specific primers for qRT-PCR is provided in Table 1. For protein analysis, cells were lysed in RIPA buffer (50 mM Tris-HCl pH 7.4, NP-40 1%, Na-deoxycholate 0.25%, NaCl 150mM, EDTA 1mM, DTT 1mM, protease cocktail 1x). Whole cell extract proteins (20 μg) were separated electrophoretically on 15-well 4–12% NuPAGE Bis-Tris polyacrylamide gels (Invitrogen). Proteins were transferred to nitrocellulose membrane (Invitrogen), blocked in 5% nonfat milk with 0.1% Tween-20 in phosphate-buffered saline. Membranes were probed with different antibodies, as indicated. Immunoreactive bands were visualized using SuperSignal West Pico chemiluminescence substrate (Thermo Scientific) and exposed to x-ray film. The antibodies used in the studies are as follows: Notch1 (sc-6014R) and AQP3 (sc-9885) from Santa Cruz; cleaved Notch1 (2421) from Cell Signaling; Hes1 (AB5702) from chemicon, involucrin (Mob270) and γ-tubulin (GTU-88) from Sigma, keratin 10 (PRB-159P), keratin 1 (PRB-149p) and loricrin (PRB-145p) from Covance. All the mRNA and protein studies were performed three times.

### Chromatin Immunoprecipitation (ChIP)

HKCs were cross-linked with 1% formaldehyde at RT for 10 min, followed by the addition of glycine to a final concentration of 125mM to terminate cross-link reaction. After washing with PBS, cells were scraped off and resuspended in SDS lysis buffer (Millipore) and sonicated by ultrasonic reactor Branson Sonifier 450 and DNA was sheared to 100-400bp. The processed chromatin was used for ChIP assays using the ChIP assay kit (Millipore) with CSL antibody (5313) from Cell Signaling. The relative amount of precipitated DNA was measured by real time PCR analysis using primers against predicted binding sites for CSL at AQP3, HES1 and HES4 promoters, respectively or a negative region located in α-satellite region which lack of CSL binding sites, and calculated after normalization to total input chromatin, according to the formula: % input = 2 ^Ct (input) – Ct (immunoprecipitation)^, Ct means cycle threshold. Enrichment fold = % input at predicted locus / % input at negative locus. All the primers used in ChIP-PCR are included in table 1. 

### siRNA Transfection

HKCs were reversely transfected with 30 nM of Silencer Select siRNAs (Invitrogen) for human AQP3 (S1521, S1523), CSL (S7251, S7253) and control (4390844) using Hiperfect reagent (Qiagen), according to manufacturer’s instructions.

### Fluorescence microscopy

8um cryostat sections of human skin were fixed with 4% paraformaldehyde in PBS at room temperature for 30 min. After permeablization with 0.1% NP-40/PBS, slides were blocked with 5% normal donkey serum/PBS for 1 hour and incubated with the primary antibodies in the blocking buffer at 4° C overnight. After 3 washes with 0.1% NP-40/PBS, slides were incubated with Alexa 488 (green)- conjugated secondary antibodies (Invitrogen) and Hoechst (Molecular Probes) for one hour. Fluorescence microscopy was carried out with Nikon TE300 inverted fluorescence microscope. The primary antibody used was the same as the one in immunodetection section.

### Alamar Blue Assay

Alamar Blue assay (Invitrogen) was used to evaluate cell proliferation. HKCs transfected with AQP3 or control siRNA. Three days post-transfection, HKCs were plated in triplicate on the 96-well collagen coated plates at a density of 1000 cells/well in 100 μl medium. When measuring proliferation rate, 10 μl of Alarma Blue reagent was added to the medium for 2 hour at 37° C. Fluorescence was monitored at 530-560 nm excitation wavelength and 590 nm emission wavelength on the VictorTM X3 Multilabel Plate Reader (Perkin Elmer, Salem, MA, USA).

### Statistical analysis

Prism software 6.0 (GraphPad software Inc.) was used to assess statistical significance of the results, employing unpaired Student’s t-test or One-way ANOVA. All quantitative real-time PCR samples were tested in triplicate and error bars represent S.E.M. P-values less than 0.05 were considered significant.

## Supporting Information

Table S1 **DNA oligonucleotide primers used in the study.**
(DOCX)
