# Supplementary material for: An Aquaporin 3-Notch1 Axis in Keratinocyte Differentiation and Inflammation
Source: PLoS One. 2013 Nov 8;8(11):e80179. doi: 10.1371/journal.pone.0080179 (PMC3832656; doi:10.1371/journal.pone.0080179)
Supplement: Table S1 — DNA oligonucleotide primers used in the study. (DOCX) [file pone.0080179.s001.docx]

**Table S1. DNA Oligonucleotide primers used in the study.**

| GENE | Forward | Reverse |
| --- | --- | --- |
|  |  |  |
| Primers for RT-PCR |  |  |
| HEY1 | TCATTTGGAGTGTTGGTGGA | CTCGCACACCATGATCACTT |
| CSL | CAAAAGTTGCACAGAAGTCATA | TGCTGCATTTCTTGGTCAC |
| TP53 | AGGCCTTGGAACTCAAGGAT | CTGAGTCAGGCCCTTCTGTC |
| HES1 | AATGACAGTGAAGCACCTCC | TCGTTCATGCACTCGCTGAA |
| AQP3 | CCTCATCCTGGTGATGTTTGG | GCCAGGTTGATGGTGAGGAA |
| CDKN1A | GATTAGCAGCGGAACAAGGA | CAACTACTCCCAGCCCCATA |
| FLG | GGGAAGTTATCTTTTCCTGTC | GATGTGCTAGCCCTGATGTTG |
| KI67 | CTGCTTGTTTGGAAGGGGT | AGCCGTACAGGCTCATCAAT |
| ITGB4 | CCCAACCACTCCTACGTGTT | GGGAGTGCTCAAAGTGAAGG |
| P16 | GCACCAGAGGCAGTAACCAT | AAGTTTCCCGAGGTTTCTCA |
| IVL | GGCCCTCAGATCGTCTCATA | CACCCTCACCCCATTAAAGA |
| KRT1 | GTTCCAGCGTGAGGTTTGTT | TAAGGCTGGGACAAATCGAC |
| KRT10 | GAAAAGCATGGGCAACTCACA | TGTCGATCTGAAGCAGGATG |
| TNFA | CCTGCTGCACTTTGGAGTGA | GAGGGTTTGCTACAACATGGG |
| CCL5 | CTCCCCATATTCCTCGGACAC | TACTCCTTGATGTGGGCACG |
| 36B4 | GCAATGTTGCCAGTGTCTGT | GCCTTGACCTTTTCAGCAAG |
|  |  |  |
| Primers for ChIP |  |  |
| AQP3 | TTAGACACGCGTCCTCTGTG | GGGTGTCTGGCCTCTAATGA |
| HES1 | CGTGTCTCTTCCTCCCATTG | CCAGGACCAAGGAGAGAGGT |
| HES4 | SimpleChIP Human Hes4 primer from | Cell Signaling #7273 |
| Negative Region | SimpleChIP Human α satellite repeat | primers from Cell Signaling #4486 |
|  |  |  |
|  |  |  |
|  |  |  |
|  |  |  |
|  |  |  |
|  |  |  |
|  |  |  |
|  |  |  |
|  |  |  |
|  |  |  |
|  |  |  |
|  |  |  |
|  |  |  |
|  |  |  |
|  |  |  |
|  |  |  |
|  |  |  |
|  |  |  |
|  |  |  |
|  |  |  |
|  |  |  |
|  |  |  |
|  |  |  |
|  |  |  |
|  |  |  |
|  |  |  |
|  |  |  |
|  |  |  |
|  |  |  |
|  |  |  |
|  |  |  |
|  |  |  |
|  |  |  |
|  |  |  |
|  |  |  |
|  |  |  |
|  |  |  |
|  |  |  |
|  |  |  |
|  |  |  |
|  |  |  |
|  |  |  |
|  |  |  |
|  |  |  |
|  |  |  |
|  |  |  |
|  |  |  |
|  |  |  |
|  |  |  |
